# Early Cognitive Dysfunction in Elderly Patients after Total Knee Arthroplasty: An Analysis of Risk Factors and Cognitive Functional Levels

**DOI:** 10.1155/2022/5372603

**Published:** 2022-02-18

**Authors:** Shengjie Ren, Fang Yuan, Shu Yuan, Chuanbo Zang, Yanan Zhang, Bao Lang

**Affiliations:** ^1^School of Anesthesiology, Weifang Medical University, Weifang 261053, China; ^2^Department of Anesthesiology, Weifang People's Hospital, Weifang 261041, China

## Abstract

**Background:**

Cognitive dysfunction after total knee arthroplasty (TKA) is very common in elderly patients. Postoperative cognitive dysfunction (POCD), as a form of cognitive dysfunction, may affect patients' short- and long-term recoveries. The identification of meaningful risk factors may help reduce the occurrence of POCD in the future.

**Objective:**

Our goal was to retrospectively investigate the risk factors for early POCD in elderly patients undergoing TKA and to further analyze the relationship between the intensity of risk factors and the level of cognitive function.

**Methods:**

The related indicators and the Montreal Cognitive Function Assessment Scale (MOCA) scores of 105 elderly patients were collected by searching the electronic case system. According to the postoperative MOCA score, patients were divided into three groups: normal group (group N), mild POCD group (group M), and severe POCD group (group S). SPSS 25.0 software was used for statistical analyses.

**Results:**

At baseline, the preoperative MOCA score was significantly different in patients with POCD (*P* ≤ 0.001), while other baseline indicators were not significantly different. In terms of changes in hemoglobin levels, statistically significant differences were observed between group M, group S, and group N (*P* = 0.039). Among inflammatory indicators, only postoperative CRP levels showed a statistically significant difference in patients with POCD (*P* = 0.041). Postoperative pain was also significantly different among the three groups (*P* = 0.009). The multivariate regression analysis revealed that a low preoperative MOCA score and severe postoperative pain were independent risk factors for mild and severe cognitive impairment, while a high postoperative CRP level was only an independent risk factor for mild cognitive impairment.

**Conclusions:**

Our study found that the level of preoperative cognitive function, postoperative CRP level, and postoperative pain were independent risk factors for POCD. Moreover, the levels of preoperative cognitive function and postoperative pain were more strongly correlated with severe POCD than postoperative CRP levels.

## 1. Introduction

With the aging of the population and the improvement of people's living standards, total knee arthroplasty (TKA) is being increasingly widely implemented in elderly patients. It successfully improves quality of life by reducing pain and improving long-term function. However, as with all surgeries, TKA may result in complications, including thromboembolism, bone-cement syndrome, anemia, cardiac arrhythmia, pain, and POCD.

POCD exerts an effect on a wide range of cognitive domains, such as memory, attention, orientation, and concentration, but a uniform definition of POCD seems to be lacking and the length of follow-up period limits comparability between studies, which also increase the challenges associated with research in this field [1]. A large number of studies have concluded that the incidence of POCD ranges from 10% to 54.3% [2]. The wild range is also related to the inconclusive diagnosis of POCD. To address this issue, the Montreal cognitive assessment (MOCA), with its high sensitivity and specificity, was developed as a tool to screen patients with mild cognitive impairment and whose MMSE is generally within the normal range [3].

POCD markedly impairs postoperative recovery, increases mortality, and increases the utilization of social and financial resources [4]. The specific mechanism of POCD remains unclear, but its related risk factors have been continuously explored. According to recent studies, in terms of the individual characteristics, age, educational level, mental health, and comorbidities may be the risk factors for POCD. In terms of surgery, hypotension, procedure time, blood loss, and a low temperature may increase the POCD incidence. In terms of anesthesia, methods of anesthesia and anesthetic drugs also affect the postoperative cognitive function. Although many studies have focused on determining the risk factors for POCD, a comprehensive analysis of risk factors in certain surgeries seems to be more useful in clinical practice. Therefore, the purpose of this study is to investigate the incidence and risk factors for early POCD and even severe POCD in elderly patients undergoing TKA.

## 2. Methods

### 2.1. Setting and Patients

This study protocol was approved by the Medical Institutional Ethics Committee of Weifang People's Hospital and was registered in the China Clinical Trial Center (ChiCTR2100049338). Patients with knee osteoarthritis (aged ≥60 years) who were scheduled to undergo TKA in general anesthesia at the Weifang People's Hospital from April 2021 to May 2021 were retrospectively included in this study. Inclusion criteria were an age greater than 60 years, patients who underwent TKA surgery, had American Society of Anesthesiologists (ASA) grades I and III, and willing and able to complete study questionnaires. Those patients who were unable to complete preoperative and postoperative MOCA evaluations and were clinically diagnosed with a neurological disorder or psychosis were excluded.

### 2.2. Anesthesia and Postoperative Treatment

All included patients were prepared for routing general anesthesia by inserting an intravenous line, and electrocardiogram, noninvasive blood pressure, pulse oximetry, and P_ET_ CO_2_ were monitored. After entering the operating room, 30 ml of 0.3% ropivacaine was used for ultrasound-guided femoral nerve block, and all patients were administered 30 *μ*g of dexmedetomidine as a premedication. Induction anesthesia was achieved with propofol (closed loop target controlled infusion under bispectral index (BIS) monitoring, initial target concentration: 3 *μ*g/ml), remifentanil (1.5 *μ*g/kg), sufentanil (10 *μ*g), and rocuronium (30 mg). The laryngeal mask was inserted when the patient was unconscious and BIS was stable at 40-60. The lungs were ventilated with 70% oxygen and controlled with a tidal volume of 6-8 ml/kg, and the ventilatory rate was 12-14 beats per minute. Respiratory parameters were adjusted to maintain a PaCO_2_ value of 35 to 40 mmHg. Propofol (closed loop target controlled infusion under BIS monitoring (40-60)) and remifentanil (0.1 *μ*g/(kg·min))was used to maintain anesthesia. When the joint cavity was opened, controlled hypotension, which was achieved by increasing the depth of anesthesia or antihypertensive drugs, was performed to reduce bleeding, and then, we maintain the target blood pressure until the end of the operation. We monitored brain oxygen saturation to avoid hypoxic ischemic injury in the brain due to hypoperfusion during anesthesia maintenance. We also used NSAIDs for pain therapy and tropisetron to reduce postoperative nausea and vomiting. Subjects were transferred to the postanesthesia care unit (PACU) after extubation. Patient-controlled intravenous analgesic (PCIA) was used for postoperative analgesia. An analgesic pump contained 170 *μ*g of dexmedetomidine and 90 *μ*g of sufentanil mixed into 100 ml of liquid, pumped at a rate of 2 ml/h. Ten milligrams of imrecoxib was orally administered twice a day in the ward.

### 2.3. Data Collection

In this study, we divided it into four time points: preoperative (T0), intraoperative (T1), and postoperative days 1 (T2) and 3 (T3). The following data were collected and recorded: (i) demographics: including sex, age, body mass index (BMI), American Society of Anesthesiologists (ASA) physical status and comorbidities (including arrhythmia, hypertension, diabetes, coronary atherosclerotic heart disease (CHD), history of stroke, and digestive system disease) and (ii) clinicopathological parameters: MOCA score was obtained prior to the operation (T0) and postoperative days 1 (T2) and 3(T3). Blood pressure was recorded as systolic blood pressure (SBP) and diastolic blood pressure (DBP), which were obtained at T0, representing the baseline level, and intraoperatively after controlling hypotension (T1). The intraoperative lowest systolic blood pressure (L-SBP) was also a cause for concern. We also calculated the magnitude of reduction in systolic blood pressure (M-SBP) and diastolic blood pressure (M-DBP) using the following specific equation: (preoperative blood pressure − controlled blood pressure)/preoperative blood pressure. Hemoglobin, hemameba, and neutrophil levels were determined at T0, T2, and T3, while C-reactive protein (CRP) levels and the erythrocyte sedimentation rate (ESR) were only determined at T0 and T3. (iii) Surgery data included the surgery duration, second surgery (Yes or No), and pattern of tourniquet use. Tourniquets can be used in two ways; one is the whole tourniquet (long-term) and the other is the tourniquet when hitting the cement (short-term). (iv) Postoperative follow-up included the degree of pain at rest (according to numeric rating scales (NRS), 1-3 was classified as mild pain, 4-6 as moderate, and 7-10 as severe) at T2 and T3.

### 2.4. Cognitive Functional Assessment and Criteria for POCD

**Figure 1:**
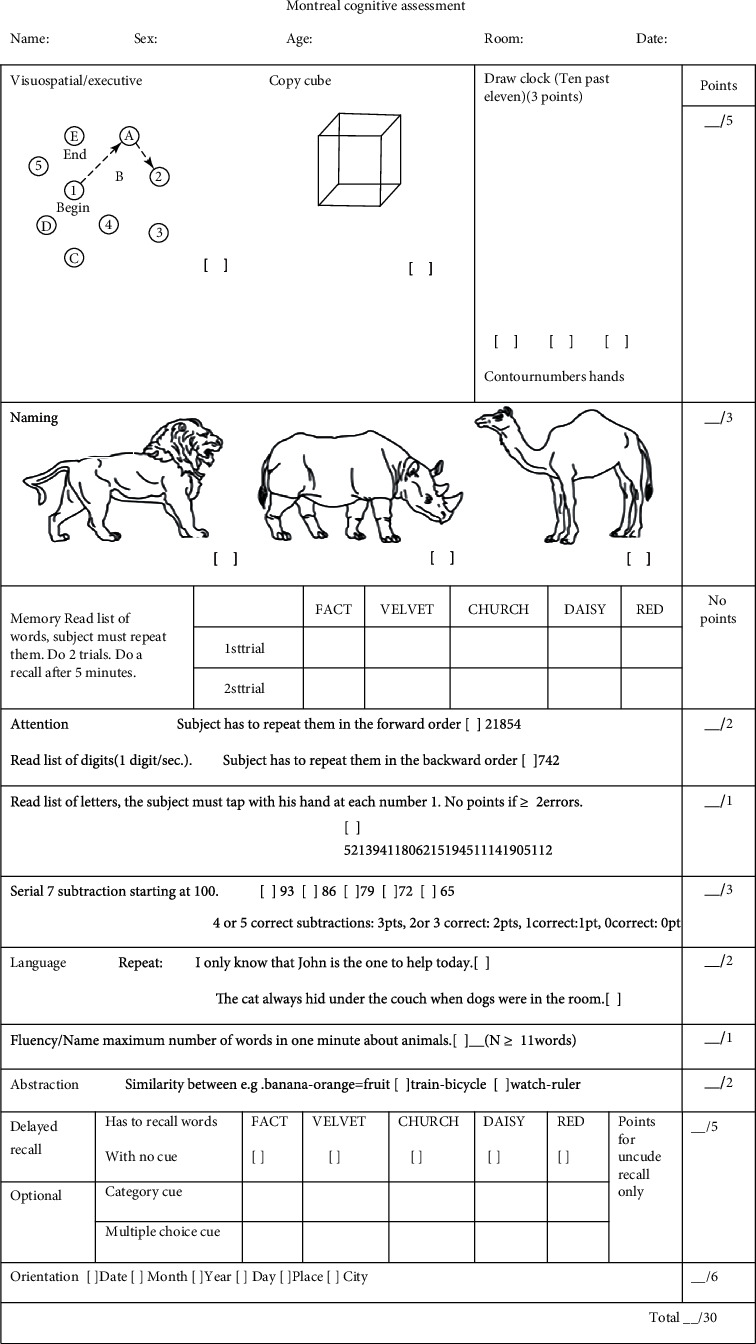
Montreal cognitive assessment (MoCA), Beijing version (in English).

The cognitive functional assessment was conducted at T0, T2, and T3 using MOCA by a trained professional. The MOCA assessment includes 16 items and 11 categories and assesses short-term memory, visuospatial abilities, executive functions, phonemic fluency, verbal abstraction, attention, concentration, working memory, language, and orientation to time and place (Figure 1). Consistent with numerous studies [5, 6], a correction for education effects was applied in present study: ≤6 years of education received a total score of +2 and 6 to 12 years of education received a total score of +1. Higher scores indicate better cognitive function. POCD was defined as a postoperative MOCA score < 26, as previously described [6–8]. Meanwhile, severe POCD was defined as a MOCA score < 20 [9, 10]. Therefore, patients were categorized into a normal group (MOCA scores ≥ 26, group N), a mild POCD group (20 ≤ MOCA scores < 26, group M), and a severe POCD group (MOCA scores < 20, group S).

### 2.5. Statistical Analysis

All data reported in this study were analyzed using SPSS 25.0 software (developed by IBM Corp). For continuous data, normally distributed data are presented as means ± standard deviations, whereas nonnormally distributed data are presented as medians (interquartile ranges [IQR]). All continuous data were analyzed using the Kruskal-Wallis test. Categorical data are presented as frequencies and proportions and were analyzed using the *χ*^2^ test or Fisher-Freeman-Halton test, as appropriate. Three pairwise comparisons were conducted using the Kruskal-Wallis test and univariate ANOVA. Relevant indicators with statistically significant differences in the previous comparison were analyzed using multifactor logistic regression. *P* < 0.05 was considered as the statistically significant level.

## 3. Result

### 3.1. General Information

One hundred five patients were able to participate in the study, and 103 patients were eventually enrolled. The remaining 2 patients were excluded because they did not have complete data. According to postoperative MOCA scores, the 103 patients were divided into three groups, group N (30 cases), group M (40 cases), and group S (33 cases). With the exception of the preoperative MOCA score, no significant differences in the baseline characteristic data were observed between the three groups, such as sex, age, BMI, SBP, DBP, hemoglobin level, ESR, CRP level, hemameba level, neutrophil counts, ASA classification, and comorbidities (including arrhythmia, hypertension, diabetes, CHD, history of stroke, and digestive system disease) (Table 1). The table depicted that the preoperative MOCA scores were significantly different between all three groups (group N: 23.83 ± 3.21, group M: 20.7 ± 2.92, group S: 15.93 ± 4.75; group N vs. group M: *P* = 0.002, group N vs. group S: *P* ≤ 0.001, group M vs. group S: *P* ≤ 0.001).

### 3.2. Intraoperative Blood Pressure

No significant difference in intraoperative blood pressure was observed between the three groups (Table 1). The L-SBP in group S (90.00 mmHg; IQR, 80.00-100.00) was numerically but not significantly lower than that in group N (100.00 mmHg; IQR, 83.75-102.75) and group M (100.00 mmHg; IQR, 90.00-113.75; *P* = 0.083). The M-SBP and M-DBP showed a tendency to increase in group N (M-SBP: 20.00%; IQR, 4.50-29.25. M-DBP: 22.50%; IQR, 13.00-35.00), group M (M-SBP: 17.00%; IQR, 7.00-26.00. M-DBP: 23.00%; IQR, 9.25-28.50), and group S (M-SBP: 24.00%; IQR, 13.00-29.00. M-DBP: 27.00%; IQR, 16.00-31.50), but no statistically significant differences were observed.

### 3.3. Postoperative Hemoglobin Level

The results shown in Table 1 indicate a significantly lower hemoglobin (T3) level in groups M (102.50 ± 15.87 g/l) and S (102.58 ± 9.11 g/l) than in group N (109.83 ± 13.29 g/l) (group N vs. group M, *P* = 0.022; group N vs. group S, *P* = 0.029). However, hemoglobin levels measured at T2 did not follow this pattern.

### 3.4. Postoperative Inflammatory Indicators

In terms of postoperative inflammatory indicators (Table 1), CRP levels in group N (44.55 ± 23.52 mg/l) at T3 were significantly reduced compared to those in group M (62.35 ± 32.31 mg/l) and group S (61.13 ± 30.24 mg/l) (*P* = 0.019 and *P* = 0.039, respectively). The ESR showed a similar trend to CRP levels, but the difference was not significant. Additionally, no significant correlation was observed for leukocyte and neutrophil counts.

### 3.5. Postoperative Pain

In the follow-up of 103 patients at T2, 30 elderly patients experienced mild pain (group N, *n* = 14 (46.7%); group M, *n* = 10 (33.3%); group S, *n* = 6 (20.0%)), 53 elderly patients experienced moderate pain (group N, *n* = 14 (26.4%); group M, *n* = 24 (45.3%); group S, *n* = 15 (28.3%)), and 20 elderly patients experienced severe pain (group N, *n* = 2 (10%); group M, *n* = 6 (30%); group S, *n* = 12 (60%)). As shown in Table 1, a significant positive correlation was observed between the level of postoperative pain and POCD (*χ*^2^ = 13.48, *P* = 0.009), whereas a significant difference in the degree of pain at T3 was not evident.

### 3.6. Surgical Information

No statistically significant differences in surgery duration were observed among groups. Regarding the use of tourniquets (Table 1), among the 19 patients who used tourniquets throughout the course, 3 (15.8%) had normal cognitive function, 9 (47.4%) had mild POCD, and 7 (36.8%) had severe POCD, but none of the differences were statistically significant (*P* = 0.363). In this study, among the 23 patients who required a second surgery, 4 (17.4%) had normal cognitive function, 11 (47.8%) had mild POCD, and 8 (34.8%) had severe POCD. Although the probability of POCD was higher during the second operation, the difference was not significant (*P* = 0.352).

### 3.7. Multivariate Regression Analysis

In the multivariate regression analysis (Table 2), the preoperative MOCA score (OR: 0.632, *P* ≤ 0.001), postoperative CRP level (OR: 1.025, *P* = 0.038), and postoperative pain at T2 (OR: 3.111, *P* = 0.023) were independent risk factors for mild POCD. Meanwhile, with the exception of postoperative CRP levels (OR: 1.028, *P* = 0.052), the preoperative MOCA score (OR: 0.396, *P* ≤ 0.001) and postoperative pain at T2 (OR: 9.348, *P* = 0.001) were also independent risk factors for severe POCD.

## 4. Discussion

After a retrospective study of 103 patients who underwent TKA, we found that preoperative MOCA scores largely determined the postoperative cognitive functional level. Severe postoperative decreases in hemoglobin levels, increased levels of an inflammatory factor (CRP), and severe pain were associated with the occurrence of POCD. The increase in CRP levels was an independent risk factor for mild POCD, while the preoperative MOCA score and postoperative severe pain were independent risk factors for mild and severe POCD. However, intraoperative blood pressure, surgery duration, tourniquet usage pattern, and times of operation had no significant correlation with POCD.

Aging was associated with global declines in network segregation, integration, and module distinctiveness, and specific declines in distinctiveness of higher-order cognitive networks; this functional network deterioration was associated with poorer cognitive performance [11]. Understandably, the elderly are at a high risk of POCD [12]. However, we did not observe a statistically significant difference in age between the three groups. This apparent difference may be based on the comparison of middle-aged and elderly groups, rather than older groups. Another potential explanation is that patients with knee osteoarthritis who need TKA are relatively healthy (no serious systemic diseases), and even a few elderly patients were >80 years old.

Although an overall decline in the cognitive function level of elderly patients has been documented, individual differences are still significant, as represented by the preoperative MOCA scores which range from 8 to 30 in our study. We evaluated the basic cognitive functional status of patients using the preoperative MOCA to reduce the interference caused by individual differences. The preoperative MOCA score was an independent predictor of mild and severe POCD in our study. This finding is consistent with recent pooled evidence on the risk factors for POCD [13]. Ghaffary et al. also found a strong relationship between the patients' basic cognition and POCD after cardiac surgery [14]. A wise approach is to screen high-risk groups for POCD through the preoperative MOCA assessment in the clinic.

Combined with the characteristics of TKA surgery (e.g., pain, blood loss), we used the controlled hypotension technology on the one hand to reduce intraoperative bleeding and on the other hand to reduce the use of a tourniquet to alleviate the pain [15]. Cerebral hypoperfusion occurring with or as a result of controlled hypotension seems inevitable. Surprisingly, no international recommendations or high quality evidence for diagnostics and treatment of neurocognitive impairment that may arise from hypotension-related hypoperfusion is available [16, 17]. Consistent with this result, this study reported the reduction in L-SBP, M-SBP, and M-DBP in patients with severe POCD, but the differences were not statistically significant. This lack of difference may be due to the existence of protective mechanisms in the brain, such as brain autoregulation function, cerebral blood flow reserve, and any factors (notably changes in arterial CO_2_ pressure) that decrease or increase cerebral blood flow (CBF) [18]. A reasonable assumption is that controlled hypotension under general anesthesia is safe, but a risk of cerebral ischemia still exists for patients with prolonged severe hypotension during surgery.

The amount of blood loss during TKA is high, and different degrees of blood oozing will occur after TKA, which lead to further increases in blood loss. Severe intraoperative and postoperative blood loss caused by TKA has always been a concern [19]. The estimation of hemoglobin loss showed a significantly better estimation of blood loss than blood volume loss formulae [20]. Blood loss, including postoperative oozing, as reflected by reduced hemoglobin levels, might render a patient susceptible to a higher risk of POCD in the present study. The conclusion that the occurrence of cognitive dysfunction is affected by intraoperative blood loss in elderly patients has consistently been confirmed [21, 22]. A deficiency in oxygen supply to the brain may result in cognitive dysfunction, which may explain the association between low hemoglobin levels and POCD [23]. A natural speculation is that transient anemia after TKA is also related to POCD. However, the authors evaluated 15,105 participants from the ELSA-Brazil Cohort Baseline and found no association between anemia and cognitive performance [24]. This difference may be caused by the use of different definitions for anemia and POCD, age of the participants, and other factors.

The general consensus is that the mechanism of postoperative cognitive dysfunction includes an inflammatory response [25, 26]. Peripheral immune cells play a role in the immune inflammatory response in the hippocampus following surgery and the inhalation of anesthesia, and the inflammatory specific biomarkers associated with the destruction of the blood-brain barrier (BBB) [27]. Chronic inflammatory disease states produced by the genetic and environmental factors also increase the risk of endothelial dysfunction with increased BBB permeability [28]. In molecular biology, microglia are important cells that maintain the balance of inflammation in the brain [29]. The increased levels of peripheral inflammatory factors induced by surgical stimuli cause microglial activation [30], which produce cytokines and result in the production of a range of inflammatory factors in areas of the central nervous system (CNS), in particular but not restricted to the hippocampus [31]. Interestingly, neuroinflammation is considered as a double-edged sword that exerts both detrimental and beneficial effects on the neurons. Inflammation-mediated neurotoxicity occurs as a consequence of microglial dysregulation and overactivation [32], whereas some data support that microglia stimulate myelin repair, remove toxic proteins from the CNS, and prevent neurodegeneration in individuals with chronic brain diseases [33, 34]. Microglial switching requires fine regulation, and more in-depth investigations are urgently needed [35]. Our study revealed that an elevated postoperative CRP level was an independent risk factor for mild POCD, but not severe POCD, which may be related to the dual effect of the inflammatory response. In addition, the result may be different when the sample size is further increased.

Postoperative pain also affected POCD in elderly patients after TKA, which was more intuitive compared with the hemoglobin level and inflammatory response. Based on clinical research using the visual analogue scale (VAS) to determine the degree of postoperative pain, POCD correlated with postoperative pain in patients who underwent abdominal surgery using different surgical methods [36]. Furthermore, untreated pain might be an important etiological factor for POCD, and it is common in elderly patients following surgery for hip fracture [37]. The mechanisms by which pain (acute and chronic pain) induces cognitive dysfunction are complex. Acute pain may aggravate postoperative cognitive dysfunction (such as memory deficits) via neurotransmitters (increased dopamine (DA) levels in the cortex and decreased acetylcholine (ACH) levels in the hippocampus) and by changing the levels of inflammatory factors [38]. The administration of effective analgesics to elderly patients might have benefits in preventing cognitive decline in some domains [39]. Compared with the effects of intravenous analgesia, the postoperative epidural analgesia decreased the systemic inflammatory response, the perceived pain, and the incidence of POCD [40, 41]. Preemptive analgesia induced by a continuous femoral nerve block may promote the recovery of early cognitive function in elderly patients following TKA [42]. The effect on the level of cognitive function is one of the indicators of choice for analgesia.

Prolonged surgery duration may lead to increased blood loss and inflammatory responses, as well as increased use of anesthetic drugs. As shown in numerous studies, the duration of the operation affects the occurrence of POCD [43]. However, no correlation between the time of operation and the occurrence of POCD was observed in our study. This result is also consistent with some other studies [44]. This finding may be due to the fact that our patients were operated on by the same surgical team, and the duration of the surgery itself did not vary substantially.

Tourniquet use has been linked to adverse outcomes resulting from intraoperative and postoperative ischemia, such as nerve paralysis, soft-tissue damage, and thromboembolism [45]. Tourniquet-related ischemia-reperfusion injury during TKA leads to altered muscle protein metabolism, endothelial dysfunction, oxidative stress, and an inflammatory response [46]. Based on advantages of the use of a tourniquet for short periods in all aspects [47], we hypothesized that the short-term use of tourniquets also promoted cognitive recovery. This research indicates that the incidence of POCD in elderly patients with full tourniquet use was higher than that in the short-term tourniquet group, but the difference was not statistically significant. Some studies also found that the patients who experienced controlled hypotension but without tourniquet use during the operation had higher MOCA scores than those patients who used tourniquets [15]. Therefore, a reduced time of tourniquet use is recommended.

Secondary surgery is quite common in elderly patients with osteoarthritis of the knee. The result illustrated that the elderly patients are more likely to develop POCD after the second surgery, but the difference was not significant. A few studies examined the relationship between a second TKA and POCD. Future studies could expand the sample size to verify these finding and to further explore the most appropriate time interval between the two operations.

Combined with surgical characteristics, we comprehensively analyzed the risk factors for POCD in elderly patients after TKA, such as tourniquet use and secondary surgery, which were not included in previous studies. The effect of secondary surgery and tourniquet use on POCD needs to be confirmed by more large-sample studies. In the clinical work about knee replacement surgery, preoperative cognitive functional level assessment maybe screen high-risk groups for POCD, and it is reasonable to reduce the occurrence of POCD by reducing inflammatory response, controlling bleeding, and reducing postoperative pain.

## 5. Conclusions

Elderly patients with TKA often suffer from different levels of POCD. In the multivariate regression analysis, the preoperative cognitive function level, increased postoperative CRP level, and severe pain were independent risk factors for mild POCD. Moreover, the preoperative cognitive function level and severe pain contributed to POCD to a greater extent than the postoperative CRP level. The postoperative hemoglobin level may be related to the occurrence of POCD. These risk factors can be used to estimate the high-risk population and severity of POCD to guide clinical practice.

## Figures and Tables

**Table 1:** Risk factor analyses of POCD.

	Group N (*n* = 30)	Group M (*n* = 40)	Group S (*n* = 33)	*P* value
*Part 1: patients' baseline data*				
Female, *n* (%)	18 (25.8)	26 (37.1)	26 (37.1)	0.245
Age (year)	66.97 ± 6.33	67.20 ± 5.71	67.18 ± 5.97	0.970
BMI (kg/m^2^)	25.71 ± 2.51	27.07 ± 3.90	26.54 ± 4.56	0.264
SBP (mmHg)	135.70 ± 22.31	140.15 ± 15.76	141.36 ± 12.19	0.404
DBP (mmHg)	86.87 ± 18.01	83.63 ± 7.49	83.76 ± 9.35	0.928
Hemoglobin (g/l)	133.03 ± 14.81	128.15 ± 14.78	130.12 ± 11.29	0.429
MoCA score	23.83 ± 3.21^ab^	20.7 ± 2.92^ac^	15.93 ± 4.75^bc^	≤0.001
ESR, mm/h (range)	11.00 (7.75, 14.50)	12.50 (9.00, 16.75)	14.00 (9.50, 17.00)	0.326
CRP, mg/l (range)	2.10 (1.47, 4.37)	2.65 (1.32, 4.55)	3.00 (1.95, 4.20)	0.701
Hemameba (∗10^9^/l)	5.50 ± 1.25	5.45 ± 1.31	5.52 ± 1.25	0.806
Neutrophils (∗10^9^/l)	3.07 ± 0.74	2.98 ± 1.01	2.88 ± 0.94	0.487
Arrhythmia, *n* (%)	1 (16.7)	3 (50)	2 (33.3)	0.795
Hypertension, *n* (%)	15 (36.6)	14 (34.1)	12 (29.3)	0.409
Diabetes, *n* (%)	3 (27.3)	5 (45.5)	3 (27.3)	0.924
CHD, *n* (%)	2 (33.3)	3 (50.0)	1 (16.7)	0.765
Stroke, *n* (%)	1 (12.5)	3 (37.5)	4 (50.0)	0.479
Digestive system disease, *n* (%)	2 (33.3)	3 (50.0)	1 (16.7)	0.765
ASAI, *n* (%)	14 (27.5)	20 (39.2)	17 (33.3)	0.994
II, *n* (%)	13 (30.2)	17 (39.5)	13 (30.2)	
III, *n* (%)	3 (33.3)	3 (33.3)	3 (33.3)	
*Part 2: intraoperative blood pressure*				
Intraoperative SBP, mmHg (range)	110.00 (100.00, 120.00)	110.00 (100.00, 130.00)	110.00 (100.00, 120.00)	0.244
Intraoperative DBP, mmHg (range)	65.00 (60.00, 71.25)	65.00 (60.00, 77.50)	65.00 (60.00, 70.00)	0.444
L-SBP, mmHg (range)	100.00 (83.75, 102.75)	100.00 (90.00, 113.75)	90.00 (80.00, 100.00)	0.083
M-SBP, % (range)	20.00 (4.50, 29.25)	17.00 (7.00, 26.00)	24.00 (13.00, 29.00)	0.169
M-DBP, % (range)	22.50 (13.00, 35.00)	23.00 (9.25, 28.50)	27.00 (16.00, 31.50)	0.381
*Part 3: hemoglobin*				
Hemoglobin (T2) (g/l)	114.90 ± 14.16	112.05 ± 16.80	113.79 ± 11.68	0.528
Hemoglobin (T3) (g/l)	109.83 ± 13.29^de^	102.50 ± 15.87^d^	102.58 ± 9.11^e^	0.039
*Part 4: inflammatory indicators*				
Postoperative ESR (mm/h)	36.02 ± 12.811	45.71 ± 19.50	44.18 ± 21.84	0.066
Postoperative CRP (mg/l)	44.55 ± 23.52^fg^	62.35 ± 32.31^f^	61.13 ± 30.24^g^	0.041
Leukocytes, ∗10^9^/l (T2)	9.34 (7.03, 11.34)	8.62 (7.74, 11,00)	8.99 (7.64, 11.16)	0.941
Leukocytes, ∗10^9^/l (T3)	6.14 (5.40, 7.45)	6.15 (5.34, 7.36)	6.63 (5.22, 7.40)	0.724
Neutrophils, ∗10^9^/l (T2)	6.54 (5.31, 9.13)	6.77 (5.31, 9.23)	7.32 (6.00, 9.20)	0.772
Neutrophils, ∗10^9^/l (T3)	4.46 (3.52, 5.51)	4.50 (3.81, 5.51)	4.45 (3.23, 5.78)	0.877
*Part 5: postoperative pain*				
Pain (T2)				
Mild, *n*(%)	14 (46.7)	10 (33.3)	6 (20.0)	0.009^∗^
Moderate, *n* (%)	14 (26.4)	24 (45.3)	15 (28.3)	
Severe, *n* (%)	2 (10.0)	6 (30.0)	12 (60.0)	
Pain (T3)				
Mild, *n* (%)	27 (31.4)	35 (40.7)	24 (27.9)	0.160
Moderate, *n* (%)	3 (18.8)	4 (25.0)	9 (56.3)	
Severe, *n* (%)	0 (0.0)	1 (100.0)	0 (0.0)	
*Part 6: surgical information*				
Surgery duration, min	59.30 ± 19.005	55.95 ± 13.832	61.18 ± 16.918	0.288
Tourniquets				
Short time, *n* (%)	27 (32.1)	31 (36.9)	26 (31.0)	0.363
Long time, *n* (%)	3 (15.8)	9 (47.4)	7 (36.8)	
Second surgery				
No, *n* (%)	26 (32.5)	29 (36.3)	25 (31.3)	0.352
Yes, *n* (%)	4 (17.4)	11 (47.8)	8 (34.8)	

Values are means ± SDs, numbers (%), or medians and ranges. Abbreviations: CRP: C-reactive protein; CHD: coronary atherosclerotic heart disease; SBP: systolic blood pressure; DBP: diastolic blood pressure; ESR: erythrocyte sedimentation rate; BMI: body mass index; ASA, American Society of Anesthesiologists; SBP: systolic blood pressure; DBP: diastolic blood pressure; L-SBP: lowest systolic blood pressure; M-SBP: magnitude of blood pressure reduction in systolic blood pressure; M-DBP: magnitude of blood pressure reduction in diastolic blood pressure. ^a^Group N vs. group M, *P* = 0.002. ^b^Group N vs. group S, *P* ≤ 0.001. ^c^Group M vs. group S, *P* ≤ 0.001. ^d^Group N vs. group M, *P* = 0.022. ^e^Group N vs. group S, *P* = 0.029. ^f^Group N vs. group M, *P* = 0.019. ^g^Group N vs. group S, *P* = 0.039. ^∗^*χ*^2^ = 13.48, *P* = 0.009.

**Table 2:** Multivariate stepwise logistical regression analysis.

		OR	Corrected OR	*P* value	Lower limit	Upper limit
Mild POCD	Preoperative MOCA	0.702	0.632	≤0.001	0.497	0.803
	Postoperative CRP	1.024	1.025	0.038	1.001	1.049
	Hemoglobin (T3)	0.959	0.957	0.094	0.909	1.008
	Pain (T2)	2.042	3.111	0.023	1.169	8.280
Severe POCD	Preoperative MOCA	0.462	0.396	≤0.001	0.281	0.559
	Postoperative CRP	1.023	1.028	0.052	1.000	1.058
	Hemoglobin (T3)	0.959	0.941	0.056	0.885	1.002
	Pain (T2)	3.845	9.348	0.001	2.539	3.442

Abbreviation: POCD: postoperative cognitive dysfunction.

## Data Availability

All the data involved in this study have been included within the article.
